# Flow cytometric reporter assays provide robust functional analysis of signaling complexes

**DOI:** 10.1016/j.jbc.2022.102666

**Published:** 2022-11-02

**Authors:** Timothy W. Muusse, Morris Y.L. Lee, Hyoyoung Kim, Marie-Odile Parat, Jeffrey D. Nanson, Bostjan Kobe, Parimala R. Vajjhala, Katryn J. Stacey

**Affiliations:** 1 School of Chemistry and Molecular Biosciences and Australian Infectious Diseases Research Centre, The University of Queensland, Brisbane, QLD, Australia; 2 School of Pharmacy, The University of Queensland, Brisbane, QLD, Australia

**Keywords:** signaling reporter assay, flow cytometry, protein-protein interaction, structure-function, lipopolysaccharide, toll-like receptor, toll-like receptor 4, myeloid differentiation response gene (88), adaptor protein, innate immunity, FSC, forward scatter, hiFCS, heat inactivated fetal calf serum, LPS, lipopolysaccharide, mEGFP, monomeric enhanced GFP, SEAP, secreted embryonic alkaline phosphatase, TIR, Toll/interleukin-1 receptor, TLR, toll-like receptor

## Abstract

Conventional assays to probe signaling protein interactions and function involve measurement of luciferase reporter expression within the bulk cell population, with lack of control over target-protein expression level. To address this issue, we have developed a rapid and robust flow cytometric assay for analysis of signaling protein function. A fluorescent reporter and fluorescent tagging of the target protein enables simultaneous assessment of protein expression and signaling within individual cells. We have applied our technique to the analysis of variants of the lipopolysaccharide receptor Toll-like receptor 4 (TLR4) and its adapter protein MyD88, using a NF-кB–responsive promoter driving mScarlet-I expression. The assay enables exclusion of nontransfected cells and overexpressing cells that signal spontaneously. Additionally, our assay allows the identification of protein variants that fail to express. We found that the assays were highly sensitive, with cells expressing an appropriate level of GFP-MyD88 showing approximately 200-fold induction of mScarlet-I by lipopolysaccharide, and we can detect subtle protein concentration-dependent effects of mutations. Importantly, the assay is adaptable to various signaling pathways.

Cell signaling requires protein interactions that mediate formation of complexes ranging from dimers to large assemblies. Molecular definition of how the signaling proteins interact to culminate in enzyme activation is paramount to understanding signaling mechanisms, their disturbance in disease states, and the potential for drug targeting. Cell-based methods for analysis of the interactions and function of signaling proteins need improvement. Most approaches involve site-directed mutagenesis and assays of signaling activity. The ideal structure-function analysis in signaling pathways would involve knock-in mutations in the endogenous locus, but this is not practical when screening multiple mutations. Instead, transient expression of variant proteins in cells lacking endogenous expression, and assessment of the induction of a pathway-specific reporter gene, such as luciferase, provides a practical approach (1, 2, 3, 4, 5, 6). However, this technique is complicated by variable transfection efficiency between samples or between experiments, variable expression levels across the cell population, protein expression above physiological levels, and possible mutation-specific effects on protein expression. Standard reporter systems that are assessed on bulk cell populations cannot resolve these problems nor control for variations in protein expression. Furthermore, when expression of the target protein is assessed, for example by Western blotting, there are no means of accurate compensation for varied expression levels, and a substantial range is usually tolerated.

Flow cytometry is the perfect technique to solve problems of current signaling reporter assays, by using a fluorescent reporter system combined with fluorescently tagged target proteins. Despite their wide use in visualizing protein localization and a range of other reporter functions, fluorescent proteins have not been exploited as reporters in mutational analysis of signaling pathways. When the target protein is fluorescently tagged, both signaling output and target protein expression level can be assessed within individual cells. This permits analysis of signaling function to be limited to those cells with an appropriate level of target protein expression, ideally as close to endogenous levels as possible. Here, we aimed to develop simple flow cytometry–based fluorescent reporter assays for studying protein interactions within the TLR4 signaling pathway, in order to validate structures that are being generated for signaling protein complexes (7, 8).

Toll-like receptors (TLRs) are innate immune receptors that detect and respond to a range of pathogen molecules as well as host danger signals. TLR4 is the receptor for bacterial lipopolysaccharide (LPS) and elicits defense against Gram-negative bacterial infections, as well as contributing to sepsis and a range of chronic inflammatory conditions (9, 10). LPS binding into the hydrophobic pocket of the TLR4 coreceptor MD-2 promotes dimerization of two TLR4-MD2-LPS subcomplexes (11, 12, 13, 14). This is presumed to lead to dimerization of the TLR4 cytosolic Toll/interleukin-1 receptor (TIR) domain and recruitment of adaptor proteins myeloid differentiation primary response gene 88 (MyD88) and MyD88 adaptor-like (MAL) (15). Ultimately, the assembly initiates kinase activity, leading to activation of transcription factors NF-кB and AP-1, and induction of proinflammatory cytokines. Following internalization of TLR4 into endosomes, signaling *via* adaptors TRAM (TRIF-related adaptor molecule) and TRIF (TIR domain-containing adaptor protein inducing interferon-β) leads to activation of transcription factor IRF3, promoting inflammation and a type-I IFN response (16, 17, 18, 19, 20). The four adaptor molecules all contain TIR domains, and their mode of interaction is starting to be elucidated with the structural characterization of assemblies reconstituted *in vitro* (7, 8). Interaction analysis in cells to validate these structures requires a reliable cellular signaling assay.

Mutational studies of TLR signaling components have previously used NF-кB–driven luciferase reporter assays on the bulk cell population (4, 5, 6, 21, 22, 23). Assessment of the levels of expressed TLR or adaptor proteins, although not always performed, has used semiquantitative Western blots or sometimes immunostaining and flow cytometry. Here, we have combined transient expression of monomeric enhanced GFP (mEGFP)-tagged TLR4 or MyD88 in HEK293 cell lines with a stably integrated NF-κB–driven mScarlet-I reporter to allow convenient single-cell analysis. While these cells may not recapitulate all the regulatory subtlety of innate immune cell signaling, the essential protein–protein interactions will be preserved and can be readily analyzed. Furthermore, HEK293 cells lack innate immune DNA sensing pathways initiated by TLR9, cGAS, and AIM2 (24, 25), enabling DNA transfection without these interfering signal outputs. We demonstrate that our dual fluorophore flow cytometric assay can be used successfully in a moderate throughput manner. This concept could be adapted for use in a wide range of signaling pathways to generate robust characterization of protein interactions.

## Results

### Generation of a fluorescent NF-κB reporter TLR4 signaling cell line with *MyD88* knock-out

**Figure 1:**
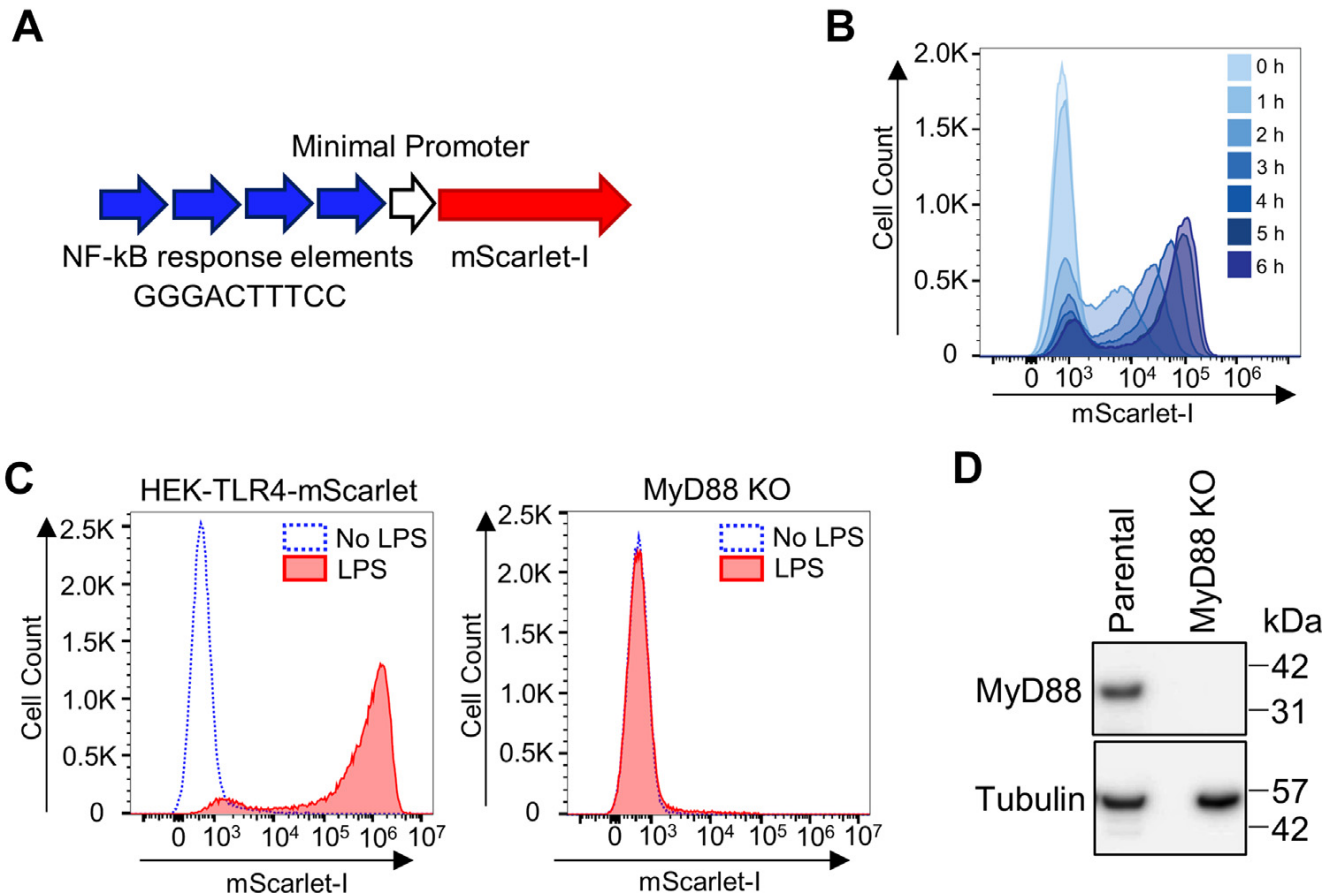
**Generation of a TLR4 signaling reporter line with a knockout of *MyD88*.***A*, NF-κB reporter construct containing four NF-κB response elements and a minimal promoter driving the mScarlet-I gene. *B*, HEK-TLR4-mScarlet cells express mScarlet-I in a time-dependent manner with LPS stimulation. Cells were treated with 100 ng/ml LPS for the stated times, fixed with 4% paraformaldehyde and then analyzed by flow cytometry. Histograms of mScarlet-I expression at the different time points are shown overlaid. *C*, CRISPR/Cas9 KO of *MyD88* in HEK-TLR4-mScarlet cells prevents LPS-induced activation of the NF-κB-mScarlet-I reporter. Responses of the TLR4-mScarlet cell line and its *MyD88* KO derivative to 16-h treatment with 100 ng/ml LPS are shown. *D*, Western blot analysis of MyD88 protein in the KO and parental cell extracts. LPS, lipopolysaccharide; TLR, toll-like receptor.

Published functional studies of transiently expressed MyD88 amino acid variants (26, 27) have been compromised by using cells that express endogenous MyD88. Thus, only spontaneous signaling upon overexpression of variants could be assessed, and not LPS-induced signaling. To allow definitive investigation of the effect of amino acid changes in the TIR domain of MyD88 on its signaling capacity, we generated a cell line containing TLR4 and a NF-κB–driven reporter plasmid but lacking MyD88 expression. We first constructed a NF-κB–driven mScarlet-I reporter plasmid (Fig. 1*A*) and stably transfected it into a HEK293 cell line that expresses TLR4, MD2, and CD14 (HEK-Blue hTLR4). A selected clone referred to as HEK-TLR4-mScarlet showed a time-dependent response to LPS (Fig. 1*B*), with a profound fluorescence shift in ∼96% of cells at 16 h after LPS treatment (Fig. 1*C*). The CRISPR/Cas9 system was then used to knock out *MyD88*. A clone was selected that lacked LPS-dependent signaling as well as MyD88 protein expression detected by Western blot (Fig. 1, *C* and *D*).

### Establishment of a highly sensitive dual fluorophore assay for functional analysis of MyD88

**Figure 2:**
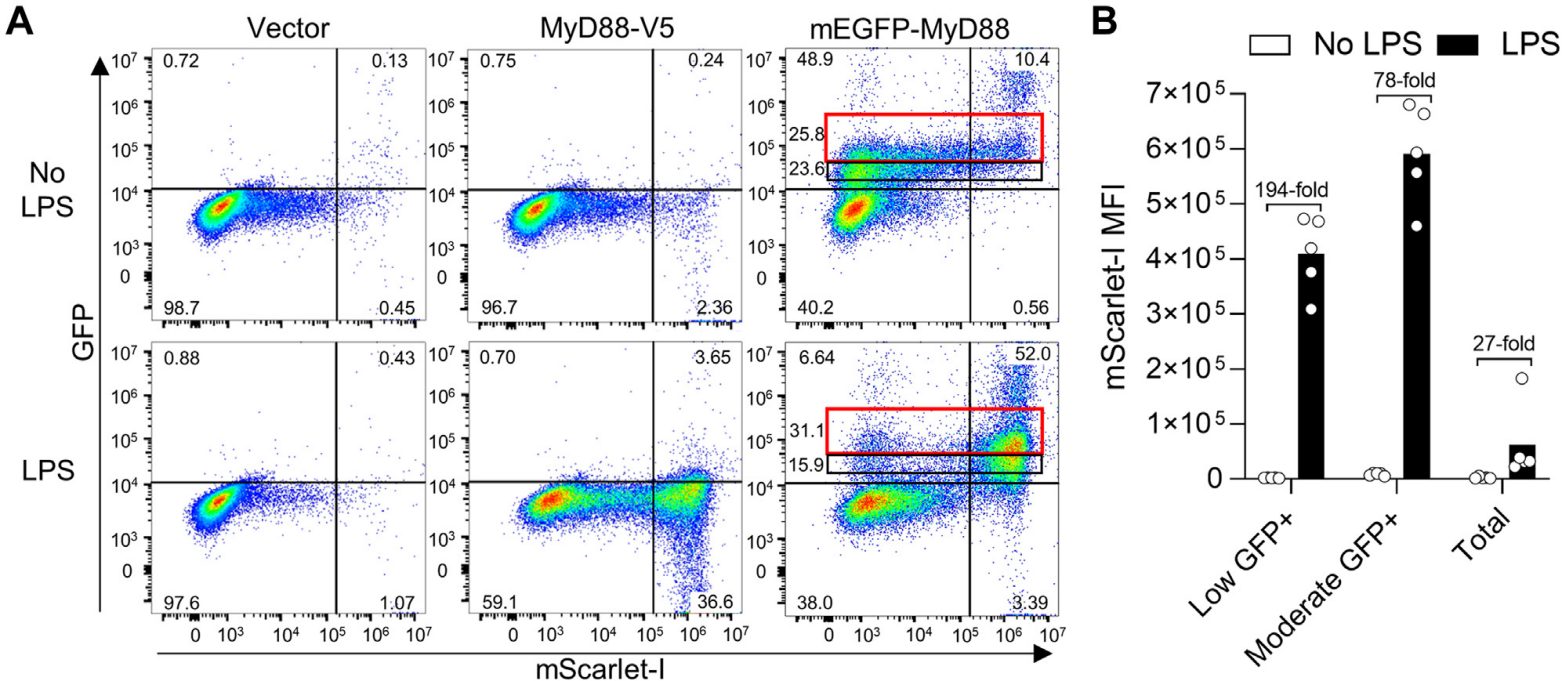
**A flow cytometric dual fluorophore assay for LPS-induced NF-κB signaling with GFP-MyD88.***A*, HEK-TLR4-mScarlet *MyD88* KO cells were transfected with either pEF6 empty vector, MyD88-V5, or GFP-MyD88. Samples were treated ± 100 ng/ml LPS and analyzed by flow cytometry. The *black* and *red* gates define two populations of GFP+ cells for analysis. Results are typical of three experiments. *B*, analysis of cells with low or moderate levels of MyD88, or the total cell population, upon LPS treatment. Data is derived from the *black* and *red* gates in panel A or was ungated for the total population. Bars show the means and data points shown are from five independent experiments (n = 5). Fold induction in response to LPS is indicated. LPS, lipopolysaccharide; TLR, toll-like receptor.

We assessed the use of HEK-TLR4-mScarlet *MyD88* KO cells for determining the signaling functionality of transfected GFP-MyD88 that is tagged on the N-terminus with mEGFP, as well as nonfluorescently tagged MyD88-V5. LPS-dependent signaling was observed for both constructs, indicated by substantial increases in mScarlet-I expression (Fig. 2*A*). A dose of 25 ng of plasmid per well was selected in prior optimization, as higher amounts of plasmid gave increased levels of spontaneous signaling. The GFP tag enabled selection of MyD88-expressing cells and exclusion of overexpressing cells. Two gates for possible analysis are shown. Both gates gave strong induction, with a reduction in basal signaling using the lower black gate (Fig. 2*B*). The black gate provided a 194-fold increase in mScarlet-I geometric mean upon LPS treatment, compared with 78-fold for the higher red gate. Ungated data gave only 27-fold induction.

### Analysis window for transient MyD88 expression is comparable to endogenous protein levels

**Figure 3:**
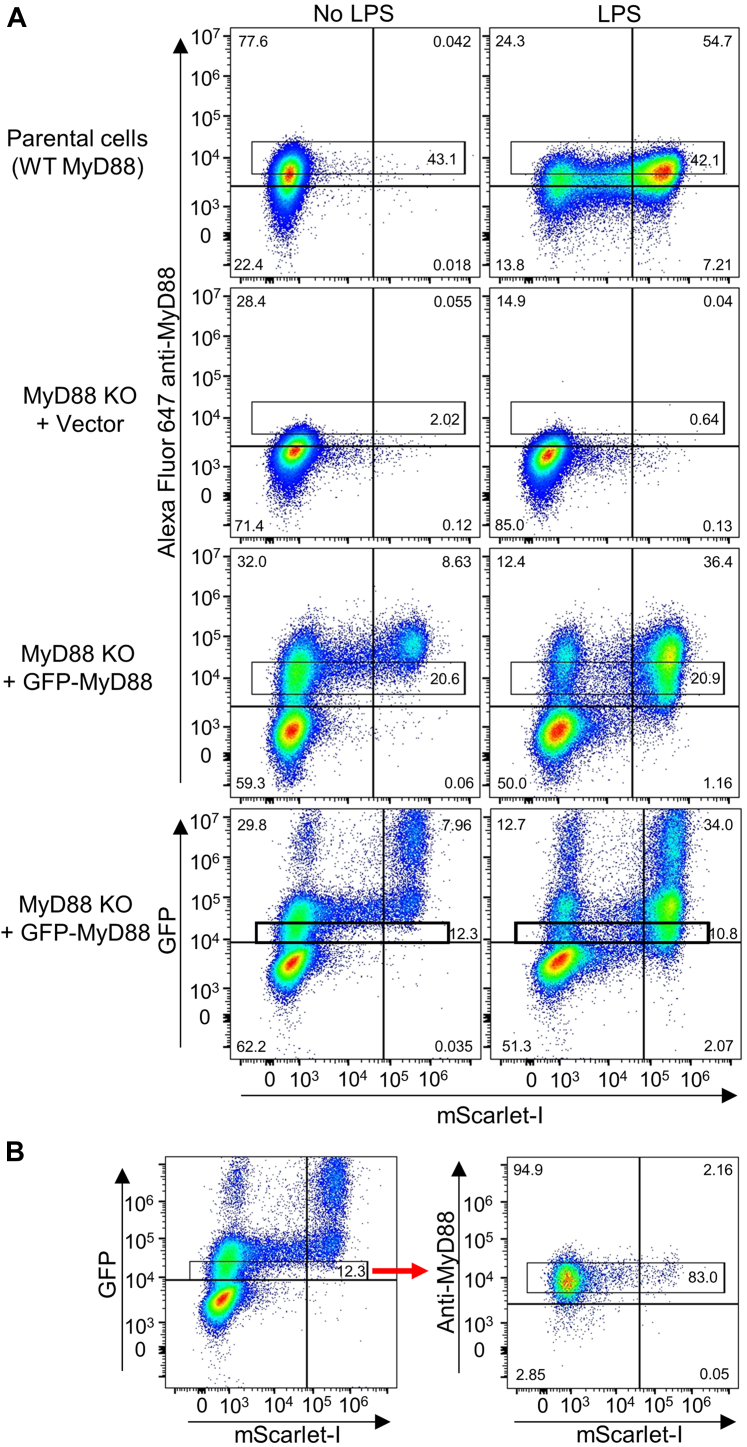
**Endogenous levels of MyD88 are comparable to transient expression in KO cells.** HEK-TLR4-mScarlet cells expressing endogenous MyD88 (parental cells) and corresponding *MyD88* KO cells with or without transiently expressed GFP-tagged MyD88 protein were treated ± 100 ng/ml LPS. Cells were immunolabeled with anti-MyD88 antibody and Alexa Fluor 647-labeled secondary antibody. *A*, the gating on Alexa Fluor 647 plots select cells clearly positive for MyD88 within the endogenous range (see also Fig. S1*A*) and below the level giving constitutive signaling. Data is representative of two experiments. *B*, analysis of data from panel A for *MyD88* KO cells transiently expressing GFP-MyD88 using a gate for low GFP shows that these cells have levels of MyD88 expression within the endogenous range. The cells falling within the gate on the left hand panel were analyzed for Alexa Fluor 647 anti-MyD88 labeling in the right hand panel. The window in the right hand panel was defined by endogenous expression levels in (A). LPS, lipopolysaccharide; TLR, toll-like receptor.

We compared the levels of MyD88 achieved by transient expression in the *MyD88* KO cells to the endogenous expression in the parental HEK-TLR4-mScarlet-I cells by immunostaining with an anti-MyD88 antibody followed by Alexa 647-labeled secondary antibody (Figs. 3*A* and S1*A*). The lower range of expression in transiently transfected cells overlaps with endogenous expression. Cells within the endogenous range of MyD88 respond appropriately to LPS (Fig. 3*A*). The upper limit of endogenous protein expression appears to be close to the threshold for ligand-independent signaling seen with immunolabeled GFP-MyD88 (Fig. 3*A*).

Interestingly, assessment of the GFP signal showed some populations with much higher GFP-MyD88 expression levels than those seen with immunolabeling (Fig. 3*A*). MyD88 overexpression does cause large aggregates in cells (8) and it is likely that this obscures the antibody epitope. Therefore, GFP-MyD88 will give more reliable representation of the true level of expression than immunolabeling. However, for proteins where the 27 kDa GFP tag interferes with protein function, the protein can be directly immunolabelled (Fig. 3) or a small epitope tag such as V5 (Fig. S1*B*) can be used.

We next examined whether the subset of GFP-MyD88–expressing cells with low level GFP signal, similar to that used previously for analysis (Fig. 2*A*), demonstrate expression at endogenous levels. GFP-MyD88–expressing cells were immunolabeled with anti-MyD88, allowing simultaneous assessment of both measures of expression and comparison to endogenous expression. The cells were first gated for low GFP expression, and then the gated cells were analyzed for the signal from anti-MyD88 immunolabeling (Fig. 3*B*). Notably, the resulting population largely fell within the gate for endogenous expression defined in Figure 3*A*. Overall, the results show that selection of the population of cells with GFP-MyD88 at a level below that which causes spontaneous signaling ensures a physiologically relevant protein level.

### Single cell analysis in *MyD88* KO cells allows evaluation of MyD88 variants

**Figure 4:**
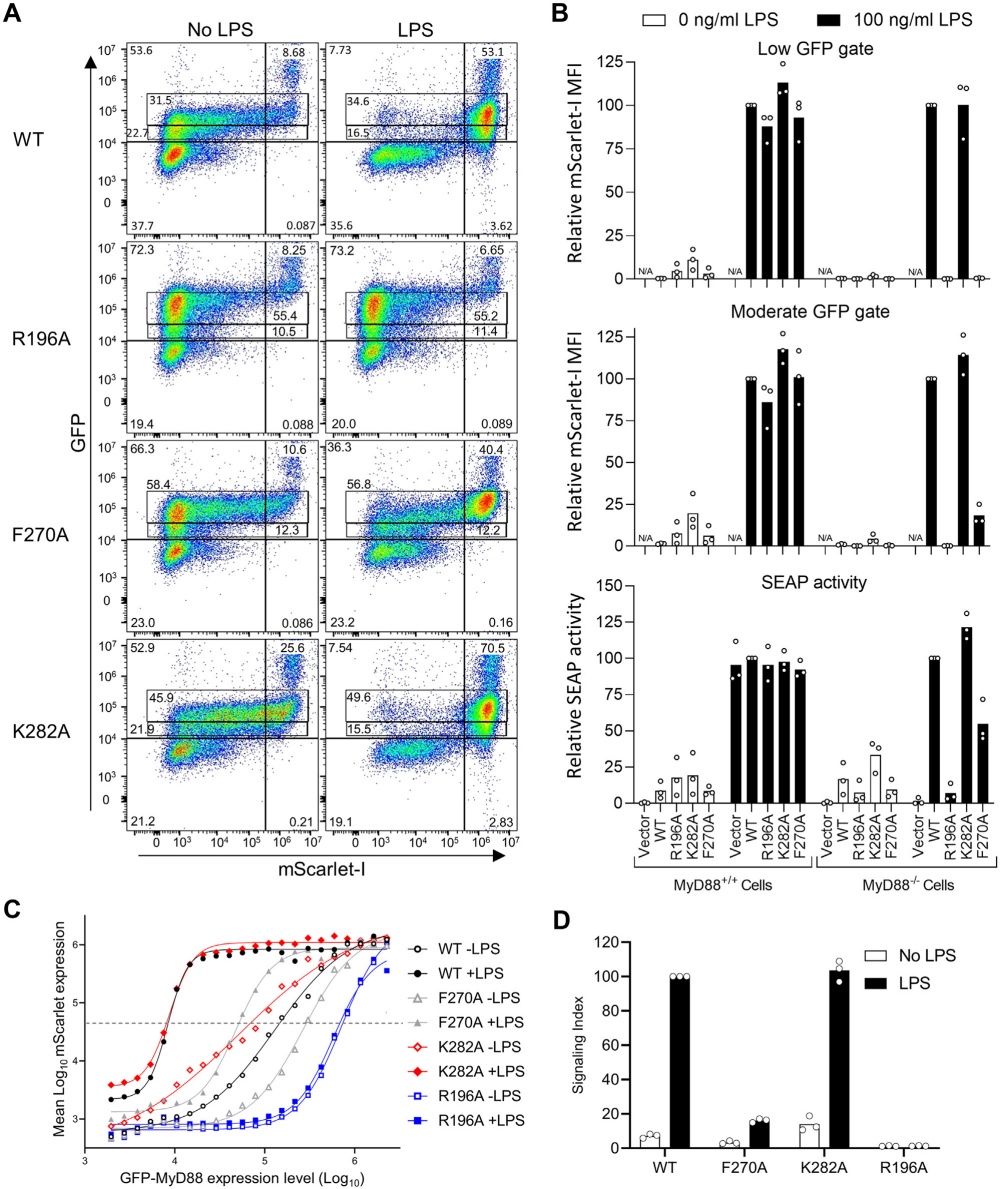
**Reconstitution of MyD88 in a KO cell line allows assessment of the function of MyD88 TIR domain variants.** HEK-TLR4-mScarlet cells with endogenous MyD88 as well as the corresponding *MyD88* KO cell line were transfected with WT GFP-MyD88 and MyD88 with amino acid changes R196A, F270A, and K282A. Cells were assessed for MyD88 expression and NF-κB activity by flow cytometric analysis of GFP and mScarlet-I expression, respectively. Concurrently, SEAP enzyme was assayed in the cell medium. *A*, primary flow cytometry data from *MyD88* KO cells shows dependence of LPS-induced mScarlet-I expression on the levels of GFP-MyD88 for WT and amino acid variants. The gates used for analysis and percentages of cells within these gates and quadrants are indicated. Results shown are typical of three experiments. *B*, single cell-based flow cytometric analysis of the geometric mean of mScarlet-I expression in cells gated for low and moderate GFP-MyD88 expression and relative SEAP activity in culture medium. Data are shown as the percentage of the WT GFP-MyD88 sample treated with 100 ng/ml LPS. Bars show the mean and data points show the results from three independent experiments (n = 3). N/A= not applicable, as vector alone samples do not fall within GFP-positive gates. *C*, a plot of the mean log(mScarlet) within log(GFP) expression bins indicates the GFP-MyD88 expression level at which signaling occurs. Sigmoidal curves were fitted to the average of duplicate samples from the experiment in panel *A*. *D*, signaling index was determined from the log(GFP) level giving half-maximal signaling. Intercepts of the *dashed line* in panel C were transformed by taking the inverse of the antilog, expressed relative to WT treated with 100 ng/ml LPS. Bars show the mean and data points show the results from three independent experiments (n = 3). LPS, lipopolysaccharide; SEAP, secreted embryonic alkaline phosphatase; TLR, toll-like receptor; TIR, Toll/interleukin-1 receptor.

Several MyD88 TIR domain mutants generated in the GFP-MyD88 construct were transfected into the reporter cell line (Fig. 4*A*). The selection of a population of cells with a defined GFP-MyD88 level facilitates a fair comparison of signaling and eliminates problems of variable transfection efficiency. Evaluation in HEK-TLR4-mScarlet *MyD88* KO cells demonstrated K282A was functional and R196A was inactive. MyD88 F270A showed loss of activity using the low gate representative of physiological expression levels (Fig. 3) but was still LPS-responsive at higher expression levels (Fig. 4*A*). This suggests that this mutation gives a reduced affinity for interactions within the signaling complex, which is overcome by higher expression.

We then compared the flow cytometry assay with the QUANTI-Blue secreted embryonic alkaline phosphatase (SEAP) reporter assay, as the cell line retains the NF-κB–driven SEAP reporter. Responses of both the parental HEK-TLR4-mScarlet and *MyD88* KO cell lines were tested. The parental cell line is competent for LPS-induced signaling, and hence the SEAP assay does not show differences in LPS response between the MyD88-transfected cells and the vector control (Fig. 4*B*). Results for the vector control samples are not shown for the flow cytometric assay as the responding cells do not fall within the GFP-positive window. The *MyD88* KO is thus an essential requirement for assessment of mutants within the full signaling pathway. Prior work has examined constitutive signaling resulting from overexpression in cells with endogenous MyD88 (26, 27). Assessed by SEAP assay, this failed to indicate the inactivity of MyD88 R196A (Fig. 4*B*). Single cell analysis demonstrated the benefits of selecting a specific MyD88 expression window to assess the LPS response. In the *MyD88* KO cells the SEAP reporter system demonstrated LPS-dependent signaling and showed R196A to be silencing and F270A to be handicapped. However, high basal levels of SEAP due to the inability to exclude overexpressing cells gave an average 9-fold SEAP induction by LPS for the WT MyD88, compared with 274-fold for the fluorescent assay with the low expression window (Fig. 4*B*).

Although use of the lower window shows the signaling capacity of MyD88 expressed at near physiological levels, it does not capture all the available information on the effects of mutations. To quantitatively express the degree of signaling handicap, we assessed the MyD88-GFP expression threshold at which signaling occurred. The whole data set for each sample was divided into 30 log_10_GFP expression levels and the average log_10_mScarlet expression determined for each. Curve fitting allowed determination of the level of MyD88-GFP giving a half-maximal response (Fig. 4*C*). The inverse anti-log of this value provides an index of signaling capacity with and without LPS (Fig. 4*D*). This signaling index indicated the elevated basal signaling from K282A and the more moderate handicap of F270A compared with the R196A null variant, with good reproducibility.

### Generation of a NF-κB–driven mScarlet-I reporter cell line

**Figure 5:**
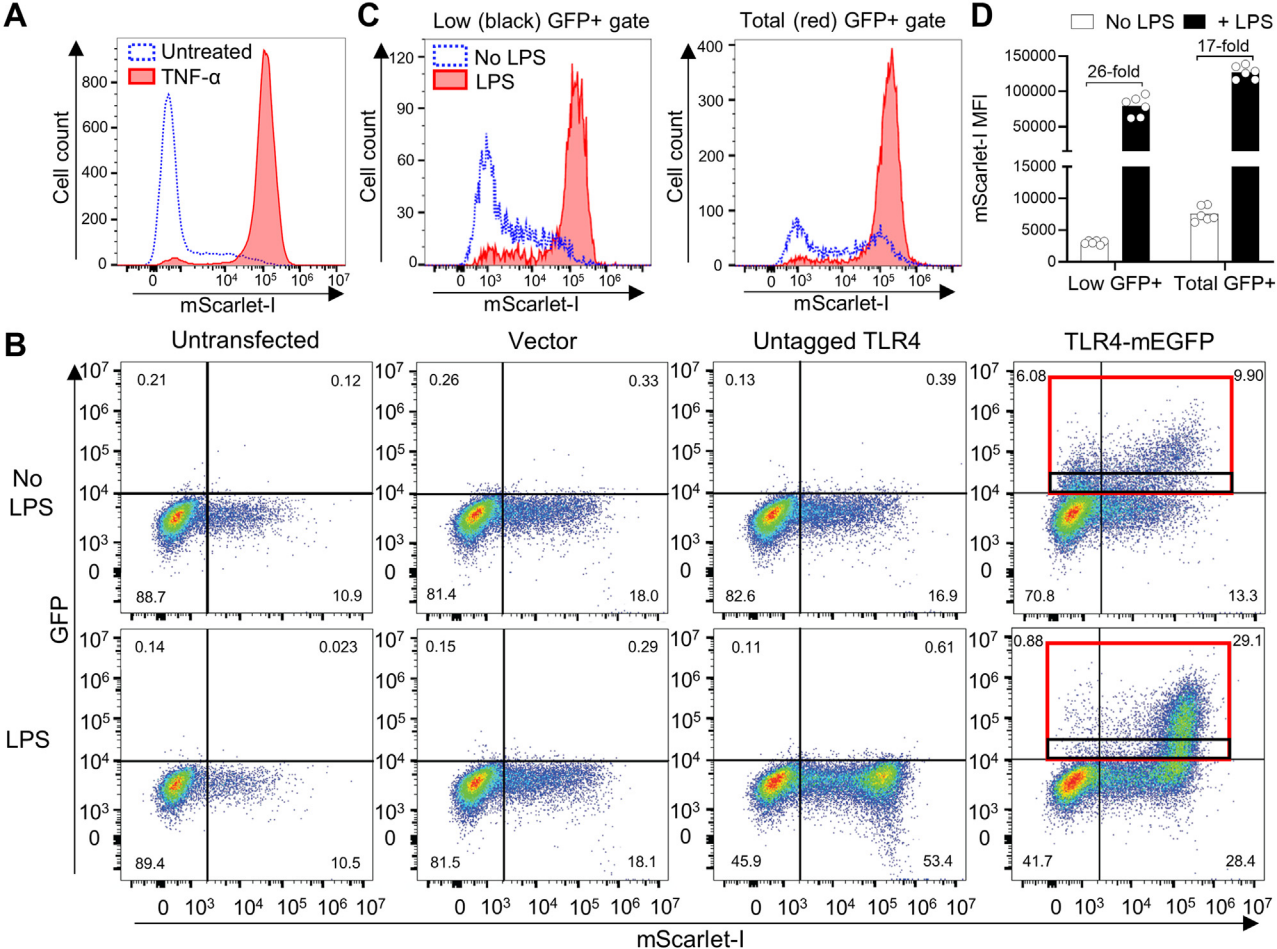
**A flow cytometric dual fluorophore assay for LPS-activated NF-κB allows analysis of cells with appropriate TLR4-GFP expression levels.***A*, generation of a reporter cell line for analyzing signaling function of TLR4 variants. Flow cytometric analysis of HEK-mScarlet cells that express CD14 and MD2, with an integrated NF-κB-mScarlet-I reporter gene after treatment with or without 100 ng/ml TNF-α for 16 h. Data is representative of three experiments. *B*, both untagged and GFP-tagged TLR4 reconstitute LPS-induced signaling in HEK-mScarlet reporter cells. Cells were either untransfected or transfected with pcDNA empty vector, untagged TLR4, or TLR4-GFP and then unstimulated or stimulated with 100 ng/ml LPS overnight and analyzed by flow cytometry. Two gating strategies are shown for TLR4-GFP: total GFP+ (*red* gate) and low GFP+ (*black* gate). Results are representative of 25 separate experiments. *C*, histograms of mScarlet-I–positive cells in the two GFP+ gates shown in panel *B*. *D*, the mScarlet-I geometric mean fluorescent intensity (MFI) in the two GFP+ gates shown in panel *B*. Bars show the means and data points shown are from six independent transfections within three experiments (n = 3). Fold induction by LPS treatment is indicated. LPS, lipopolysaccharide; TLR, toll-like receptor.

To assess the effect of mutations in the TIR domain of TLR4 on its signaling capacity, we stably transfected the NF-κB-mScarlet-I reporter plasmid into a HEK293 cell line that expresses the TLR4 coreceptors CD14 and MD2 but lacks TLR4 (HEK-Blue MD2-CD14 cells). The resulting HEK-mScarlet cell line was generated from a cell clone that showed detectable mScarlet-I expression in ∼4% of untreated cells and in ∼96% of cells post-stimulation with TNF-α, an NF-κB stimulus (28) (Fig. 5*A*).

### Establishment of a dual fluorophore assay for functional analysis of TLR4

Upon transfection with untagged TLR4, treatment with LPS led to a substantial increase in the population of mScarlet-I–expressing cells (Fig. 5*B*). The lack of LPS response in untransfected and vector-transfected cells confirmed TLR4-dependence. Some LPS-independent mScarlet-I–expressing cells were seen in all samples, with basal mScarlet-I expression increasing upon chemical transfection compared to untransfected cells. This suggests that a stress response to transfection triggers a small NF-κB response. HEK-mScarlet cells transiently transfected with a plasmid expressing TLR4 tagged with C-terminal mEGFP (TLR4-GFP) demonstrated successful LPS-dependent TLR4 activation (Fig. 5*B*), as cells that were TLR4-GFP–positive responded to LPS with a substantial shift in mScarlet-I fluorescence. The level of TLR4 required for functional signaling appears to be quite low, as some cells below the TLR4-GFP detection limit also show LPS-induced expression of mScarlet-I (Fig. 5*B*).

The overall TLR4-GFP expression increased with LPS treatment (Fig. 5*B*), due to the NF-κB–responsive CMV promoter driving the TLR4-GFP gene (29, 30). This could be avoided by use of a different promoter such as EF-1α but does not affect the ability to analyze TLR4 function. Notably, in the absence of LPS stimulation, cells with the highest levels of TLR4-GFP were predominantly mScarlet-I positive. Use of the *MyD88* KO cell line demonstrated that constitutive mScarlet-I expression in a minority of cells is partially due to an MyD88-independent stress response to chemical transfection but predominantly due to an MyD88-dependent response to TLR4 overexpression (Fig. S2). The latter may be due to a nonphysiological TLR4 aggregation recruiting MyD88, or alternatively, TLR4 dimerization forced by high expression may partially overcome the handicap of any TIR domain mutations. In either case, use of flow cytometry conveniently allows these cells to be excluded from analysis.

Results obtained by gating for a population of uniform low TLR4 expression levels (black gate, Fig. 5*B*) were compared to a gate including all GFP-positive cells (red gate). The low expression gate removes much of the LPS-independent signaling and had greater fold induction with LPS than the red gate (26-fold *versus* 17-fold) (Fig. 5*C*). Overall, the assay enables analysis of TLR4 signaling in cells with a defined TLR4-GFP expression level, excluding nonexpressing cells and cells with overly high TLR4-GFP expression where the mScarlet-I reporter expression is independent of LPS.

### Comparison of bulk population and single-cell assays for signaling

**Figure 6:**
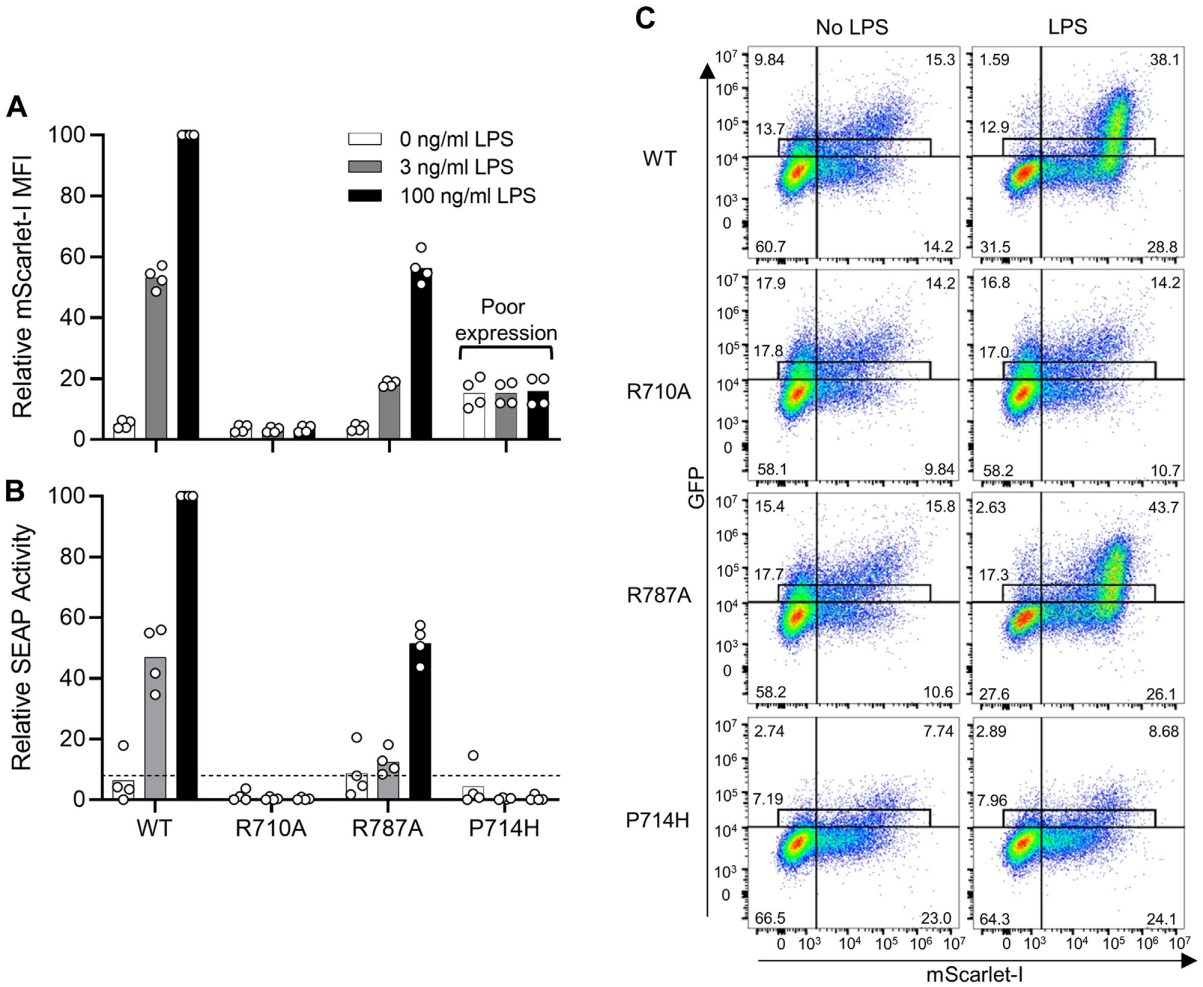
**The fluorescent reporter assay allows convenient analysis of TLR4 variant signaling with simultaneous assessment of TLR4 expression.** HEK-mScarlet reporter cells were transfected with TLR4-GFP and then either left untreated or treated with 3 ng/ml or 100 ng/ml LPS. WT TLR4-GFP and the TLR4 TIR domain mutants R710A, R787A, and P714H were tested. Cells in each experiment were assessed for NF-κB activity by both flow cytometry for mScarlet-I expression and SEAP assay of culture medium for SEAP reporter. Data are shown as the percentage of the WT TLR4-GFP sample treated with 100 ng/ml LPS. Bars show the means and data points shown are from four independent transfections within two experiments. *A*, single cell-based flow cytometric analysis of the geometric mean of NF-κB–induced mScarlet-I expression in cells gated for low TLR4-GFP. *B*, analysis of NF-κB–induced SEAP reporter enzyme activity in the culture medium. *Dotted line* denotes LPS-treated empty vector–transfected cells. *C*, primary flow cytometry data for panel *A* shows variation in TLR4-GFP expression (y-axis) and mScarlet-I expression (x-axis) for WT and mutant TLR4-GFP stimulated with or without 100 ng/ml LPS. The gate used for analysis in panel *A* and percentages of cells within this gate and quadrants are indicated. Results shown are typical of four independent transfections. LPS, lipopolysaccharide; SEAP, secreted embryonic alkaline phosphatase; TIR, Toll/interleukin-1 receptor; TLR, toll-like receptor.

We assessed several TLR4 TIR domain mutants using our mScarlet-I flow cytometry assay and SEAP reporter assay. In both assay systems, the TLR4 TIR domain mutation R787A decreased NF-κB activity by 40-50%, whereas R710A was completely inactive (Fig. 6, *A* and *B*). The results with R710A are consistent with previous publications using a luciferase reporter system (4, 22), whereas R787A has not been tested previously in isolation. The nonconservative substitution P714H showed no activity in the SEAP assay but gave low constitutive mScarlet-I expression with substantial error. However, looking at the primary flow cytometry data shows immediately that P714H is very poorly expressed in comparison to WT, R710A, and R787A constructs (Fig. 6*C*). This suggests that P714H is misfolded and unstable. P714H did not show up as spontaneously signaling in the SEAP assay, as the few TLR4-GFP-expressing cells with NF-κB activity were only a small fraction of the total cells. Overall, this demonstrates the robust results obtained with the fluorescent reporter assay, the importance of assessing the protein expression and excluding constructs with inadequate expression, and the convenience of fluorescent tagging for this purpose.

## Discussion

We have developed an innovative flow cytometric assay for analysis of protein function in signaling pathways. This technique provides substantial improvement in reliability, convenience, and information content, compared to the methods generally used in the field. The use of fluorescent reporters allows both protein expression levels and signaling activity to be determined concurrently within a single cell, without the need for staining, enzyme assays, or further sample processing. Importantly, flow cytometry allows the determination of signaling activity in cell populations with defined protein expression levels. This feature allows exclusion of untransfected cells from analysis and eliminates problems of variation in transfection efficiency between samples, protein overexpression leading to spontaneous signaling, and variable expression level between cells within the population.

The usual current practice for structure-function analysis of signaling proteins is to use a luciferase reporter gene with a pathway-specific promoter. This technique has been utilized for analysis of signaling of a wide range of proteins including steroid hormone receptors, G protein–coupled receptors, transcription factors, and innate immune receptors including TLRs (1, 2, 3, 31, 32, 33, 34). Like SEAP, luciferase activity gives an average result for the cell population. The problem of variable transfection efficiency between samples is generally tackled by normalization to a cotransfected construct with a constitutive promoter driving a *Renilla* luciferase reporter (32). Differences in protein expression levels that are intrinsic to the specific mutants being assessed are more difficult to control for. Some studies do not detail protein expression, while inferring functional effects of mutations (1). Expression is most commonly assessed by Western blotting (4, 5, 21, 22), which is generally semiquantitative (35). Furthermore, as there is no way to normalize results using this data, modest or even substantial differences in expression are generally ignored. We have solved these problems using fluorescent tagging of the protein under investigation and single-cell analysis. Flow cytometry enables exclusive analysis of ligand-specific responses, as untransfected cells and those with high expression and constitutive signaling activity can be excluded.

In previous TLR structure-function publications, protein expression and activity analyses have been performed separately, utilizing either immunofluorescent flow cytometry or Western blot to determine protein expression and NF-κB–driven luciferase or SEAP assay to evaluate TLR activity (4, 5, 6, 14, 21, 22, 23, 26, 36, 37, 38, 39, 40, 41, 42, 43, 44, 45, 46). Our analysis of WT and mutant TLR4 protein expression immediately highlights constructs that do not express well, likely due to misfolding or structural disruptions that destabilize the protein. Here, the P714H TLR4 mutant gave poor expression levels. P714H is the human counterpart of the TLR4 P712H mutation of C3H/HeJ mice, found to be responsible for their lack of response to LPS (47). Structure-function studies using transfected P714H have not previously noted deficient expression of this construct, using Western blotting and immunostaining of epitope-tagged TLR4 (6, 21, 22, 48). Although DNA sequencing showed the promoter intact, P714H showed consistently low expression across nine experiments, including data presented here. Assessment of GFP levels may be a good indicator of appropriately folded protein, whereas Western blot or immunostaining for short epitope tags will include misfolded aggregated proteins. For P714H, the small number of cells falling within the analyzed window of TLR4 expression do show somewhat elevated basal NF-κB activity. Although we considered that this could be due to the unfolded protein response that is known to elicit NF-κB signaling (49, 50), the proportion of mScarlet-positive cells in the absence of LPS is substantially reduced in the *MyD88* KO (Fig. S2). This suggests that P714H may have low constitutive signaling due to MyD88 recruitment to protein aggregates. The combination of low TLR4 expression and basal NF-κB activity is consistent with protein misfolding, aggregation, and degradation.

MyD88 is a central molecule in TLR signaling. To our knowledge, published work has not achieved reconstitution of the TLR4 pathway with MyD88 in a *MyD88* KO cell line to allow a highly sensitive response to LPS and appropriate study of mutants (26, 27). MyD88-dependent signaling has been reconstituted in *MyD88* KO immortalized mouse BMM-like cell lines; however, comprehensive studies of MyD88 variants have not been done (27, 51). By analyzing cells with MyD88 levels that were demonstrated to be physiologically relevant (Fig. 3), we obtained a minimum 194-fold induction of the reporter gene by LPS, providing sensitive detection of the effect of MyD88 variants. Signaling can be assessed at various levels of MyD88 expression to identify variants that still function but have lower affinity interactions. Alternatively, the threshold of protein expression at which signaling occurs can be used as an index showing functional handicap due to mutation. Key to the success of our signaling model is the use of *MyD88* KO cells, as the presence of the WT protein compromises analysis of the mutant proteins. Published work has generally analyzed constitutive LPS-independent signaling in response to over-expressed MyD88, in the presence of endogenous WT MyD88 expression (26, 52). In an alternative approach, one group has used expression of a dominant negative MyD88 TIR domain fragment that can inhibit LPS-induced signaling in cells with an intact TLR4 pathway (42). That style of analysis would also benefit from adaption to a fluorescent assay to control for protein expression levels.

One drawback of our technique is the need for a functioning fluorescently tagged construct. Fluorescent protein tags are generally 25 to 30 kDa and could sterically hinder normal interactions and function, although we have not so far encountered a problem in tagging of TLR signaling components. Screening of both N- and C-terminal tags and a variety of lengths of flexible linkers may be necessary. The fluorophore chosen should avoid the tendency to oligomerize and because this is not well characterized for all fluorophores, we selected the monomeric EGFP mutant (53). However, if tagging with a fluorescent protein is problematic, we showed that immunolabeling of the native protein or smaller epitope tags is possible. The caveats to this are accessibility of the epitopes when proteins are complexed, variable loss of protein during fixation and permeabilization, and increased assay complexity. Thus, where possible, genetically-encoded fluorophore tags are preferred.

The combination of fluorescent reporter output and fluorescently tagged target proteins can be used in a diverse array of experimental investigations to mimic realistic signaling conditions. It allows the transition from whole population assays and separate protein expression analysis to a single cell–based system for both. This may be especially beneficial for selecting appropriate protein expression levels in those pathways that normally function with very low endogenous protein levels. Addition of further fluorophores could expand analytical potential to look at combinations of mutants in several different proteins or two different reporter constructs. This assay offers substantial advantages over existing practices in the determination of biologically relevant protein interaction interfaces. Once an interaction model has been developed, the role of critical residues should ideally be confirmed in cells with the native signaling pathway. The most feasible system for studying TLR4 signaling complexes is immortalized macrophages from gene KO mice, although this would require retroviral transduction. This is a labor-intensive procedure, emphasizing the value of our technique for initial screening in HEK293 cells that are more readily manipulated.

## Experimental procedures

### Plasmids

A single point mutation (A207K) was introduced into EGFP in the plasmid pEGFP-N1 to prevent dimerization (53), generating mEGFP. The sequence encoding YFP in pcDNA3-TLR4-YFP (Addgene plasmid #13018, http://n2t.net/addgene:13018; RRID:Addgene 13018) was replaced with mEGFP to form the pcDNA3-hTLR4-GFP plasmid. Full-length MyD88 was cloned into pEF6 with an N-terminal mEGFP tag, with a linker (GGGGS)_3_ inserted between mEGFP and MyD88 to form the pEF6-GFP-MyD88 plasmid. The plasmid expressing MyD88-V5 has previously been described (8). To make the pNF-κB-mScarlet-I reporter plasmid (7), the cDNA encoding luciferase in the pNF-κB-Luc plasmid (Stratagene) was replaced with the cDNA encoding mScarlet-I, which was amplified by PCR from Lck-mScarlet-I expression plasmid (54) (Addgene plasmid #98821, http://n2t.net/addgene:98821; RRID:Addgene_98821). TLR4 and MyD88 mutants were generated *via* Quikchange (Stratagene) or Q5 (New England Biolabs) site-directed mutagenesis. All plasmids were verified by automated DNA sequencing (Australian Genome Research Facility). Endotoxin was removed from plasmid DNA using Triton X-114 (55).

### Cell culture

HEK-Blue hMD2-CD14 and HEK-Blue hTLR4 cells were obtained from InvivoGen. All cells were maintained in Dulbecco’s modified Eagle’s medium (DMEM) with 4.5 g/l glucose (GIBCO), 110 mg/l sodium pyruvate, supplemented with Glutamax-1 and 10% heat inactivated fetal calf serum (hiFCS; GIBCO), 50 U/ml penicillin, and 50 μg/ml streptomycin (Life Technologies), hereafter referred to as complete DMEM medium. Cells were grown in a humidified incubator at 37 °C with 5% CO_2_. All cell lines were periodically maintained in respective selective antibiotics (InvivoGen).

### Generation of reporter cell lines

The pNF-κB-mScarlet-I construct was chemically transfected into both HEK-Blue hMD2-CD14 and HEK-Blue hTLR4 cells using Lipofectamine 2000. Cotransfection of pEF6 plasmid into the HEK-Blue MD2-CD14 cells allowed selection with blasticidin, followed by single-cell cloning and screening ± 100 ng/ml TNF-α (PeproTech). This generated the HEK-Blue hMD2-CD14 NFκB-mScarlet-I cell line, termed here as HEK-mScarlet. The generated HEK-Blue hTLR4 NFκB-mScarlet-I cell line has been described previously (7).

### CRISPR knock out of *MyD88*

The CRISPR/Cas9 system was utilized to knock out *MyD88*, as described previously (7). Single-cell clones were screened with or without ultrapure *Escherichia coli* LPS 0111:B4 (Cat. tlrl-3pelps, InvivoGen) (100 ng/ml LPS) treatment for mScarlet-I expression on a Cytoflex S flow cytometer. A Western blot was run for all handicapped cell lines to determine the level of MyD88 protein, using anti-MyD88 antibody (D80F5, Cell Signaling Technology).

### Cell transfection and treatment for analysis of TLR4 and MyD88 mutants

For transfections, 64,000 cells were plated per well in a 96-well plate in 100 μl of complete DMEM medium lacking antibiotics and transfected approximately 5 h later. Transfection complexes were prepared using DNA to Lipofectamine 2000 ratio of 1 μg:2.5 μl, according to the manufacturer’s instructions (ThermoFisher Scientific). For transfection of hTLR4 plasmids, 200 ng of plasmid was transfected into cells. For MyD88 expression plasmids, 25 ng of plasmid complexed with 0.25 μl of Lipofectamine 2000 was transfected into cells. The transfection complexes were added to cells and the plate was centrifuged for 10 min at 700*g* to increase transfection efficiency (56) and incubated overnight. Medium was then replaced with fresh complete DMEM medium with 5% hiFCS and incubated for 6 h. Cells were left unstimulated or stimulated with 3 ng/ml or 100 ng/ml LPS overnight and then cells were analyzed by flow cytometry, and where stated, the medium was analyzed using the SEAP reporter assay. For transfections in a 12-well plate, 250,000 HEK-TLR4-mScarlet-I *MyD88* KO cells were plated in 1 ml of complete DMEM medium lacking antibiotics and transfected the next day. Transfections were done as described above with 400 ng of DNA.

### Immunostaining MyD88 and V5 tag

Cells were harvested in PBS and fixed with 4% paraformaldehyde for 15 to 30 min at room temp, permeabilized in PBS with 0.1% bovine serum albumin, 0.2% hiFCS, 0.1% saponin, and 0.1% NaN_3_, blocked in PBS with 0.1% bovine serum albumin, 1% hiFCS, and 0.1% NaN_3_ and then stained with rabbit anti-MyD88 antibody at 1/20,000 dilution (ab199247, Abcam) or rabbit anti-V5 antibody at 1/2000 dilution (D3H3Q, Cell Signaling Technology) overnight at 4 °C. Samples were washed in block solution. The secondary antibody goat anti-rabbit Alexa Fluor 647 F(ab')2 fragment (A21246, Life Technologies) was applied at 1/1500 dilution and incubated at room temp for 1 h. Samples were washed in block solution and resuspended in block solution for analysis.

### Flow cytometry

Samples were run on a BD Cytoflex S flow cytometer. GFP was detected using excitation at 488 nm and emission at 525 nm (525/40), and mScarlet-I was detected using excitation at 561 nm and emission at 585 nm (585/42). Gains were set to 100 for both channels. Alexa Fluor 647 was detected using excitation 638 nm and emission at 660 nm (660/20). Forward scatter (FSC) threshold was set to 80,000 and a minimum of 50,000 to 60,000 events were recorded; 100,000 events were recorded for immunolabeled samples. Data was analyzed using FlowJo 10.7.2. Samples were first gated to exclude debris and select live cells using a side scatter-area *versus* FSC-area plot and then gated for single cells on a FSC-width *versu*s FSC-area plot (Fig. S3). Single cells were then viewed as GFP/Alexa Fluor 647 *versus* mScarlet-I plots. Compensation of the mScarlet-I signal out of the GFP channel was done at 0.8 to 1.5%. Empty vector control transfections determined the threshold for TLR4-GFP or GFP-MyD88 expression, and cells expressing low levels without constitutive signaling were gated for analysis. Geometric mean for mScarlet-I fluorescence of the GFP+ gate was normalized to that of the WT treated with 100 ng/ml LPS to determine the NF-κB activity (% WT +LPS).

To generate the signaling index, compensated MyD88-GFP and mScarlet values for singlet events in all samples were acquired from FlowJo software. The log_10_GFP expression levels were assigned into 30 different bins using Power BI. The mean Log_10_mScarlet value was determined for each GFP bin, allowing curve fitting using duplicate samples within each experiment (Sigmoidal, 4PL, X is log(concentration) in GraphPad Prism9). To obtain the threshold value of MyD88-GFP which permitted signaling, for each sample, the value of log_10_GFP at which the log_10_mScarlet was equivalent to the half-maximal value for the WT + LPS sample was determined. The inverse anti-log of this value provided an index of signaling potential that can be compared with the WT protein.

### SEAP reporter assay

For each sample, 5 μl of medium was transferred to a 384-well plate and then 45 μl of QUANTI-BlueSolution (InvivoGen) was added. The absorbance at 640 nm was read at 5 min intervals for 90 min on a Clariostar microplate reader (BMG Labtech) set to 37 °C. The rate of the enzymatic reaction within the linear portion was determined. The data was normalized to the activity of WT samples treated with 100 ng/ml LPS.

## Data availability

All data are available in the main text. Distribution of cell lines requires negotiation with InvivoGen who generated the parental lines we started with, but plasmids can be supplied to recreate the final cell lines.

## Supporting information

This article contains supporting information (7).

## Conflict of interest

The authors declare that they have no competing interests.

## References

[1] Morris G., Stoychev S., Naicker P., Dirr H.W., Fanucchi S. (2018). The forkhead domain hinge-loop plays a pivotal role in DNA binding and transcriptional activity of FOXP2. J. Biol. Chem..

[2] Bhat R.A., Stauffer B., Komm B.S., Bodine P.V.N. (2007). Structure–Function analysis of secreted frizzled-related protein-1 for its Wnt antagonist function. J. Cell Biochem..

[3] Jones E.M., Lubock N.B., Venkatakrishnan A.J., Wang J., Tseng A.M., Paggi J.M. (2020). Structural and functional characterization of G protein–coupled receptors with deep mutational scanning. eLife.

[4] Xu Y., Tao X., Shen B., Horng T., Medzhitov R., Manley J.L. (2000). Structural basis for signal transduction by the Toll/interleukin-1 receptor domains. Nature.

[5] Ronni T., Agarwal V., Haykinson M., Haberland M.E., Cheng G., Smale S.T. (2003). Common interaction surfaces of the toll-like receptor 4 cytoplasmic domain stimulate multiple nuclear targets. Mol. Cell Biol..

[6] Nunez Miguel R., Wong J., Westoll J.F., Brooks H.J., O'Neill L.A., Gay N.J. (2007). A dimer of the Toll-like receptor 4 cytoplasmic domain provides a specific scaffold for the recruitment of signalling adaptor proteins. PLoS One.

[7] Clabbers M.T.B., Holmes S., Muusse T.W., Vajjhala P.R., Thygesen S.J., Malde A.K. (2021). MyD88 TIR domain higher-order assembly interactions revealed by microcrystal electron diffraction and serial femtosecond crystallography. Nat. Commun..

[8] Ve T., Vajjhala P.R., Hedger A., Croll T., DiMaio F., Horsefield S. (2017). Structural basis of TIR-domain-assembly formation in MAL- and MyD88-dependent TLR4 signaling. Nat. Struct. Mol. Biol..

[9] Akira S., Uematsu S., Takeuchi O. (2006). Pathogen recognition and innate immunity. Cell.

[10] Balka K.R., De Nardo D. (2019). Understanding early TLR signaling through the Myddosome. J. Leukoc. Biol..

[11] Shimazu R., Akashi S., Ogata H., Nagai Y., Fukudome K., Miyake K. (1999). MD-2, a molecule that confers lipopolysaccharide responsiveness on Toll-like receptor 4. J. Exp. Med..

[12] Kim H.M., Park B.S., Kim J.I., Kim S.E., Lee J., Oh S.C. (2007). Crystal structure of the TLR4-MD-2 complex with bound endotoxin antagonist Eritoran. Cell.

[13] Nagai Y., Akashi S., Nagafuku M., Ogata M., Iwakura Y., Akira S. (2002). Essential role of MD-2 in LPS responsiveness and TLR4 distribution. Nat. Immunol..

[14] Schromm A.B., Lien E., Henneke P., Chow J.C., Yoshimura A., Heine H. (2001). Molecular genetic analysis of an endotoxin nonresponder mutant cell line: a point mutation in a conserved region of MD-2 abolishes endotoxin-induced signaling. J. Exp. Med..

[15] Park B.S., Song D.H., Kim H.M., Choi B.S., Lee H., Lee J.O. (2009). The structural basis of lipopolysaccharide recognition by the TLR4-MD-2 complex. Nature.

[16] Yamamoto M., Sato S., Hemmi H., Uematsu S., Hoshino K., Kaisho T. (2003). TRAM is specifically involved in the Toll-like receptor 4-mediated MyD88-independent signaling pathway. Nat. Immunol..

[17] Ermolaeva M.A., Michallet M.C., Papadopoulou N., Utermohlen O., Kranidioti K., Kollias G. (2008). Function of TRADD in tumor necrosis factor receptor 1 signaling and in TRIF-dependent inflammatory responses. Nat. Immunol..

[18] Pobezinskaya Y.L., Kim Y.S., Choksi S., Morgan M.J., Li T., Liu C. (2008). The function of TRADD in signaling through tumor necrosis factor receptor 1 and TRIF-dependent Toll-like receptors. Nat. Immunol..

[19] Chang M., Jin W., Sun S.C. (2009). Peli1 facilitates TRIF-dependent Toll-like receptor signaling and proinflammatory cytokine production. Nat. Immunol..

[20] Hacker H., Karin M. (2006). Regulation and function of IKK and IKK-related kinases. Sci. STKE.

[21] Ding Y., Qiu Y., Zou L., Tan Z., Dai J., Xu W. (2015). Three conserved MyD88-recruiting TLR residues exert different effects on the human TLR4 signaling pathway. Immunol. Res..

[22] Bovijn C., Ulrichts P., De Smet A.S., Catteeuw D., Beyaert R., Tavernier J. (2012). Identification of interaction sites for dimerization and adapter recruitment in Toll/interleukin-1 receptor (TIR) domain of Toll-like receptor 4. J. Biol. Chem..

[23] Rallabhandi P., Bell J., Boukhvalova M.S., Medvedev A., Lorenz E., Arditi M. (2006). Analysis of TLR4 polymorphic variants: new insights into TLR4/MD-2/CD14 stoichiometry, structure, and signaling. J. Immunol..

[24] Amalfi S., Molina G.N., Bevacqua R.J., Lopez M.G., Taboga O., Alfonso V. (2020). Baculovirus transduction in mammalian cells is affected by the production of type I and III interferons, which is mediated mainly by the cGAS-STING pathway. J. Virol..

[25] Thul P.J., Akesson L., Wiking M., Mahdessian D., Geladaki A., Ait Blal H. (2017). A subcellular map of the human proteome. Science.

[26] Vyncke L., Bovijn C., Pauwels E., Van Acker T., Ruyssinck E., Burg E. (2016). Reconstructing the TIR side of the myddosome: a paradigm for TIR-TIR interactions. Structure.

[27] Nagpal K., Plantinga T.S., Sirois C.M., Monks B.G., Latz E., Netea M.G. (2011). Natural loss-of-function mutation of myeloid differentiation protein 88 disrupts its ability to form Myddosomes. J. Biol. Chem..

[28] Schütze S., Wiegmann K., Machleidt T., Krönke M. (1995). TNF-induced activation of NF-kappa B. Immunobiology.

[29] Dorsch-Häsler K., Keil G.M., Weber F., Jasin M., Schaffner W., Koszinowski U.H. (1985). A long and complex enhancer activates transcription of the gene coding for the highly abundant immediate early mRNA in murine cytomegalovirus. Proc. Natl. Acad. Sci. U. S. A..

[30] Pan R.Y., Xiao X., Chen S.L., Li J., Lin L.C., Wang H.J. (1999). Disease-inducible transgene expression from a recombinant adeno-associated virus vector in a rat arthritis model. J. Virol..

[31] Ji H., Li Y., Liu Z., Tang M., Zou L., Su F. (2020). Quantitative evaluation of the transcriptional activity of steroid hormone receptor mutants and variants using a single vector with two reporters and a receptor expression cassette. Front. Endocrinol..

[32] Ao D., Xia P., Jiang S., Chen N., Meurens F., Zhu J. (2019). Comparative transcriptome analysis of TLR8 signaling cells revealed the porcine TLR8 specific differentially expressed genes. Dev. Comp. Immunol..

[33] Pinskey J.M., Franks N.E., McMellen A.N., Giger R.J., Allen B.L. (2017). Neuropilin-1 promotes Hedgehog signaling through a novel cytoplasmic motif. J. Biol. Chem..

[34] Qiu H., Qin A., Cheng T., Chim S.M., Smithers L., Chen K. (2021). A missense mutation sheds light on a novel structure–function relationship of RANKL. J. Cell Physiol..

[35] Butler T.A.J., Paul J.W., Chan E.-C., Smith R., Tolosa J.M. (2019). Misleading westerns: common quantification mistakes in western blot densitometry and proposed corrective measures. Biomed. Res. Int..

[36] Hughes M.M., Lavrencic P., Coll R.C., Ve T., Ryan D.G., Williams N.C. (2017). Solution structure of the TLR adaptor MAL/TIRAP reveals an intact BB loop and supports MAL Cys91 glutathionylation for signaling. Proc. Natl. Acad. Sci. U. S. A..

[37] Kawai T., Takeuchi O., Fujita T., Inoue J.-i., Mühlradt P.F., Sato S. (2001). Lipopolysaccharide stimulates the MyD88-independent pathway and results in activation of IFN-regulatory factor 3 and the expression of a subset of lipopolysaccharide-inducible genes. J. Immunol..

[38] Khor C.C., Chapman S.J., Vannberg F.O., Dunne A., Murphy C., Ling E.Y. (2007). A Mal functional variant is associated with protection against invasive pneumococcal disease, bacteremia, malaria and tuberculosis. Nat. Genet..

[39] Lin Z.J., Lu J., Zhou W.H., Shen Y.Q. (2012). Structural insights into TIR domain specificity of the bridging adaptor mal in TLR4 signaling. PLoS One.

[40] Mansell A., Brint E., Gould J.A., O'Neill L.A., Hertzog P.J. (2004). Mal interacts with tumor necrosis factor receptor-associated factor (TRAF)-6 to mediate NF-kappaB activation by toll-like receptor (TLR)-2 and TLR4. J. Biol. Chem..

[41] Miggin S.M., Pålsson-McDermott E., Dunne A., Jefferies C., Pinteaux E., Banahan K. (2007). NF-kappaB activation by the Toll-IL-1 receptor domain protein MyD88 adapter-like is regulated by caspase-1. Proc. Natl. Acad. Sci. U. S. A..

[42] Ohnishi H., Tochio H., Kato Z., Orii K.E., Li A., Kimura T. (2009). Structural basis for the multiple interactions of the MyD88 TIR domain in TLR4 signaling. Proc. Natl. Acad. Sci. U. S. A..

[43] Fitzgerald K.A., Palsson-McDermott E.M., Bowie A.G., Jefferies C.A., Mansell A.S., Brady G. (2001). Mal (MyD88-adapter-like) is required for Toll-like receptor-4 signal transduction. Nature.

[44] Enokizono Y., Kumeta H., Funami K., Horiuchi M., Sarmiento J., Yamashita K. (2013). Structures and interface mapping of the TIR domain-containing adaptor molecules involved in interferon signaling. Proc. Natl. Acad. Sci. U. S. A..

[45] Facchini F.A., Zaffaroni L., Minotti A., Rapisarda S., Calabrese V., Forcella M. (2018). Structure–activity relationship in monosaccharide-based toll-like receptor 4 (TLR4) antagonists. J. Med. Chem..

[46] Kwon H.-K., Patra M.C., Shin H.-J., Gui X., Achek A., Panneerselvam S. (2019). A cell-penetrating peptide blocks Toll-like receptor-mediated downstream signaling and ameliorates autoimmune and inflammatory diseases in mice. Exp. Mol. Med..

[47] Poltorak A., He X., Smirnova I., Liu M.Y., Van Huffel C., Du X. (1998). Defective LPS signaling in C3H/HeJ and C57BL/10ScCr mice: mutations in Tlr4 gene. Science.

[48] Medvedev A.E., Piao W., Shoenfelt J., Rhee S.H., Chen H., Basu S. (2007). Role of TLR4 tyrosine phosphorylation in signal transduction and endotoxin tolerance. J. Biol. Chem..

[49] Ebstein F., Poli Harlowe M.C., Studencka-Turski M., Krüger E. (2019). Contribution of the unfolded protein response (UPR) to the pathogenesis of proteasome-associated autoinflammatory syndromes (PRAAS). Front. Immunol..

[50] Smith J.A. (2018). Regulation of cytokine production by the unfolded protein response; implications for infection and autoimmunity. Front. Immunol..

[51] Nagpal K., Plantinga T.S., Wong J., Monks B.G., Gay N.J., Netea M.G. (2009). A TIR domain variant of MyD88 adapter-like (Mal)/TIRAP results in loss of MyD88 binding and reduced TLR2/TLR4 signaling. J. Biol. Chem..

[52] Loiarro M., Volpe E., Ruggiero V., Gallo G., Furlan R., Maiorino C. (2013). Mutational analysis identifies residues crucial for homodimerization of myeloid differentiation factor 88 (MyD88) and for its function in immune cells. J. Biol. Chem..

[53] Zacharias D.A., Violin J.D., Newton A.C., Tsien R.Y. (2002). Partitioning of lipid-modified monomeric GFPs into membrane microdomains of live cells. Science.

[54] Chertkova A.O., Mastop M., Postma M., Bommel N., Niet S., Batenburg K.L. (2017). Robust and bright genetically encoded fluorescent markers for highlighting structures and compartments in mammalian cells. BioRxiv.

[55] Stacey K.J., Young G.R., Clark F., Sester D.P., Roberts T.L., Naik S. (2003). The molecular basis for the lack of immunostimulatory activity of vertebrate DNA. J. Immunol..

[56] Stacey K.J., Idris A., Sagulenko V., Vitak N., Sester D.P. (2016). Methods for delivering DNA to intracellular receptors. Met. Mol. Biol..

