# Supplementary material for: Flow cytometric reporter assays provide robust functional analysis of signaling complexes
Source: J Biol Chem. 2022 Nov 2;298(12):102666. doi: 10.1016/j.jbc.2022.102666 (PMC9747584; doi:10.1016/j.jbc.2022.102666)
Supplement: Supporting information [file mmc1.pdf]

Flow cytometric reporter assays provide robust functional analysis of signaling complexes

Timothy W. Muusse, Morris Y. L. Lee, Hyoyoung Kim, Marie-Odile Parat, Jeffrey D. Nanson, Bostjan Kobe, Parimala R. Vajjhala, Katryn J. Stacey

Supporting Information

Figure S1

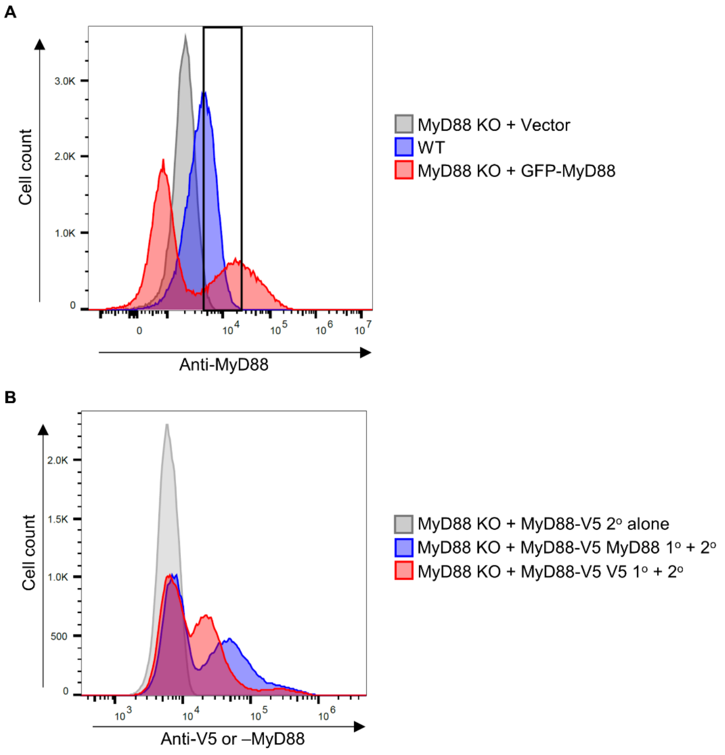

Supporting Data Fig. 1 Transient MyD88 expression can be detected by immunostaining and is comparable to endogenous MyD88. (**A**) Data from Fig. 3A is replotted as a histogram to show the reactivity of anti-MyD88 antibody to parental HEK-TLR4-mScarlet cells containing endogenous MyD88 ("WT") compared to either non-expressing or GFP-MyD88-expressing HEK-TLR4-mScarlet *MyD88* KO cells. Anti-MyD88 antibody was detected with an Alexa Fluor 647-conjugated secondary antibody. Despite optimization of antibody concentration, some background staining with this antibody is seen in the *MyD88* KO cells transfected with vector alone, that is higher than in the non-expressing cells within the GFP-MyD88 sample. However, the window of expression shown (as used in Fig. 3) contains the overlapping MyD88 expression for endogenous and transiently expressed protein that is detectable above the vector alone negative control. (**B**) A V5-epitope tag provides an alternative measure of MyD88 detection. HEK-TLR4-mScarlet *MyD88* KO cells were transfected with GFP-MyD88 or MyD88-V5. Cells were immunolabelled with anti-V5 or anti-MyD88 antibodies and Alexa Fluor 488-labelled secondary antibody or with secondary antibody alone. We previously found immunolabelling for MyD88-V5 to be of poor sensitivity (1), but results with an optimized staining procedure now suggest V5-tagging provides a versatile strategy for cases where a GFP tag is not suitable and a direct primary antibody is not available.

Figure S2

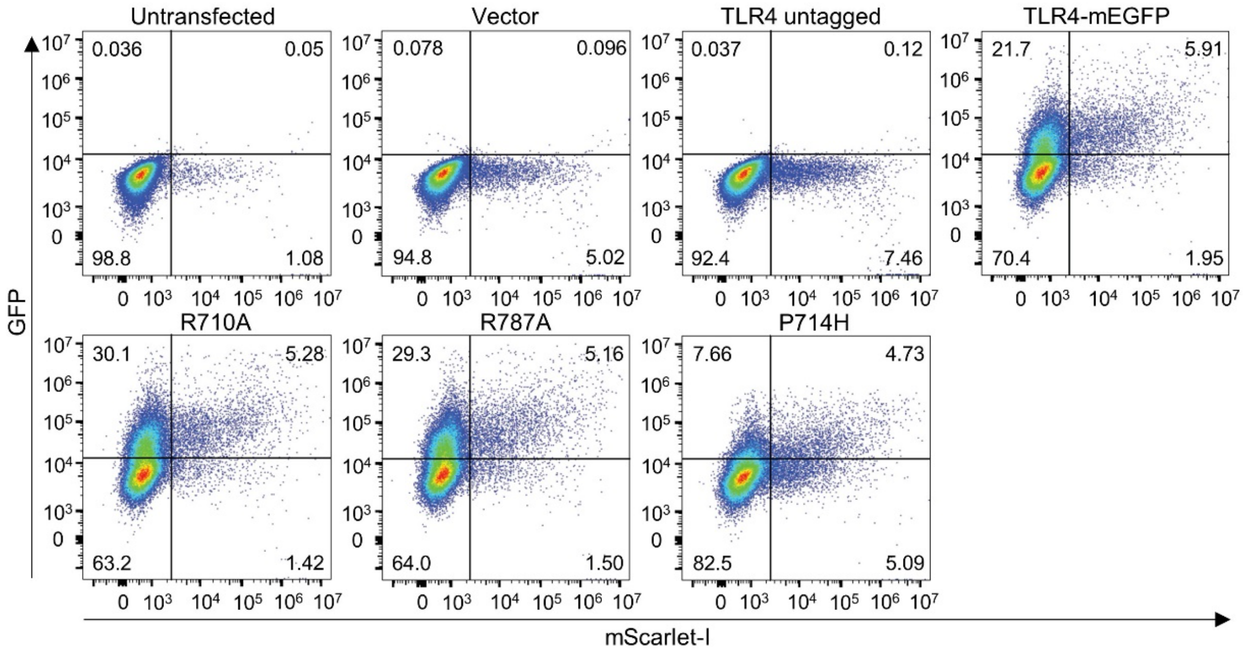

Supporting Data Fig. 2 Knock-out of *MyD88* limits the LPS-independent NF-κB activation seen at high TLR4-GFP. HEK-TLR4-mScarlet MyD88 KO cells were left untransfected or transfected with pcDNA vector, untagged TLR4, WT TLR4-GFP or the TLR4 TIR domain mutants R710A, R787A and P714H. Cells were analyzed by flow cytometry for GFP and mScarlet-I expression as in Fig. 6C but without LPS treatment. The percentage of cells within each quadrant are indicated. Results shown are typical of 3 experiments for WT and R710A, and 2 experiments for other mutants. The results of Figure 5C using HEK-mScarlet cells show that cells highly expressing WT TLR4 tend to be mScarlet-I-positive without LPS treatment. However, here in the absence of MyD88, the majority of GFP-positive cells remain mScarlet-I-negative. This suggests that a high level of TLR4 expression causes spontaneous MyD88-dependent signaling. Results further emphasize the poor expression of MyD88 P714H.

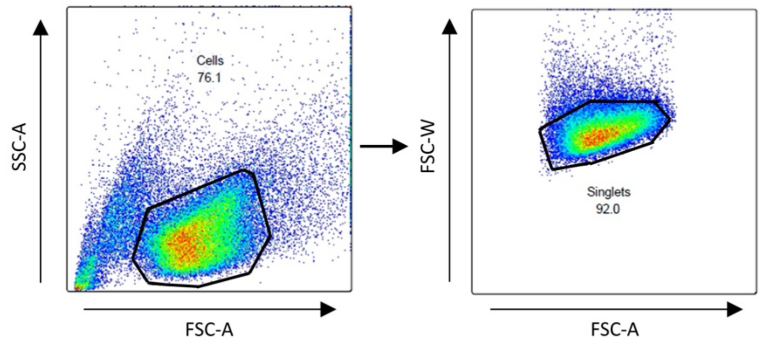

Supporting Data Fig. 3 Preliminary gating in flow cytometry analysis. Events collected in flow cytometry were gated to remove debris and dead cells using a plot of side scatter-area (SSC-A) vs forward scatter-area (FSC-A). This was followed by selection of single cells and removal of cell doublets using a plot of FSC-width vs FSC-A.

1. Clabbers, M. T. B., Holmes, S., Muusse, T. W., Vajjhala, P. R., Thygesen, S. J., Malde, A. K., Hunter, D. J. B., Croll, T. I., Flueckiger, L., Nanson, J. D., Rahaman, M. H., Aquila, A., Hunter, M. S., Liang, M., Yoon, C. H., Zhao, J., Zatsepin, N. A., Abbey, B., Sierecki, E., Gambin, Y., Stacey, K. J., Darmanin, C., Kobe, B., Xu, H., and Ve, T. (2021) MyD88 TIR domain higher-order assembly interactions revealed by microcrystal electron diffraction and serial femtosecond crystallography. *Nat Commun* **12**, 2578
